# Erratum to: Micronized/ultramicronized palmitoylethanolamide displays superior oral efficacy compared to nonmicronized palmitoylethanolamide in a rat model of inflammatory pain

**DOI:** 10.1186/s12974-016-0595-6

**Published:** 2016-05-31

**Authors:** Daniela Impellizzeri, Giuseppe Bruschetta, Marika Cordaro, Rosalia Crupi, Rosalba Siracusa, Emanuela Esposito, Salvatore Cuzzocrea

**Affiliations:** Department of Biological and Environmental Sciences, University of Messina, Viale Ferdinando Stagno D’Alcontres, no 31, Messina, 98166 Italy; Manchester Biomedical Research Centre, Manchester Royal Infirmary, University of Manchester, Manchester, Oxford Rd, Manchester, M13 9WL UK

## Erratum

The authors would like to issue an erratum for this article [1], and would like to declare the following competing interests which we inadvertently failed to include in our original publication. The authors would like to apologise for this omission.

## Competing interests

Dr. Salvatore Cuzzocrea, researcher on the study team, is co-inventor on patent WO2013121449 A8 (Epitech Group SpA) which deals with compositions and methods for the modulation of amidases capable of hydrolysing N-acylethanolamines useable in the therapy of inflammatory diseases. Moreover, Dr. Cuzzocrea is also a co-inventor with Epitech group on the following patents:

1. EP 2 821 083
2. MI2014 A001495
3. 102015000067344

No other authors have competing interests.
